# Continuous local minocycline delivery from PLA nanosphere-coated dental implants with long-term antibacterial activity and biocompatibility

**DOI:** 10.1039/d6ra05856f

**Published:** 2026-09-24

**Authors:** Napossorn Patiyananuwat, Chalaisorn Thanapongpibul, Panarin Chinavinijkul, Bongkot Phumkrachai, Chinnadit Ngamwongronnachai, Salunya Tancharoen, Norased Nasongkla

**Affiliations:** a Department of Biomedical Engineering, Faculty of Engineering, Mahidol University Phutthamonthon Nakhon Pathom 73170 Thailand norased.nas@mahidol.ac.th; b Department of Chemistry and Center of Excellence for Innovation in Chemistry, Faculty of Science, Mahidol University Bangkok 10400 Thailand; c National Laboratory Animal Center, Mahidol University Phutthamonthon Nakhon Pathom 73170 Thailand; d Department of Pharmacology, Faculty of Dentistry, Mahidol University Bangkok 10400 Thailand salunya.tan@mahidol.ac.th

## Abstract

Peri-implant infections remain a major cause of dental implant failure and are often associated with bacterial colonization and biofilm formation on implant surfaces. This study aimed to develop and evaluate minocycline (MC)-coated dental implants for local antibiotic delivery and infection prevention. Drug-free poly(d,l-lactic acid) (PLA) nanospheres and free MC were co-deposited onto the implant surface. The coated implants were evaluated for drug release, antibacterial activity, biocompatibility, and osseointegration. *In vitro* drug release studies demonstrated continuous MC release for at least 12 weeks, with concentrations remaining above the minimum inhibitory concentration (MIC) of *Porphyromonas gingivalis*. Antibacterial activity was confirmed by bacterial immersion and agar diffusion assays, showing inhibition of *P. gingivalis* growth for up to 12 weeks. Hemocompatibility and cytocompatibility evaluations revealed negligible hemolysis and high cell viability, indicating good *in vitro* biocompatibility. *In vivo* intracutaneous irritation and skin sensitization studies performed according to ISO 10993 standards demonstrated no evidence of irritation or delayed hypersensitivity reactions. Furthermore, a 12-week implantation study in rabbits demonstrated no adverse clinical signs, inflammatory responses, foreign body reactions, or tissue necrosis. Histological examination revealed direct bone-to-implant contact, normal bone marrow architecture, and the absence of fibrous encapsulation in both uncoated and MC-coated implants, indicating successful osseointegration. Collectively, the results demonstrate that MC-coated dental implants provided prolonged local antibiotic delivery and long-term antibacterial activity while maintaining biocompatibility and osseointegration. The PLA nanosphere coating approach may facilitate the development of drug-eluting dental implants for localized delivery of therapeutic agents to support peri-implant tissue health and reduce the risk of implant-associated infections.

## Abstract 2

A PLA-NS/MC layer-by-layer coating enables prolonged local minocycline delivery from titanium dental implants, maintaining antibacterial activity against *P. gingivalis* for more than 12 weeks while preserving biocompatibility and osseointegration.

## Graphical abstract

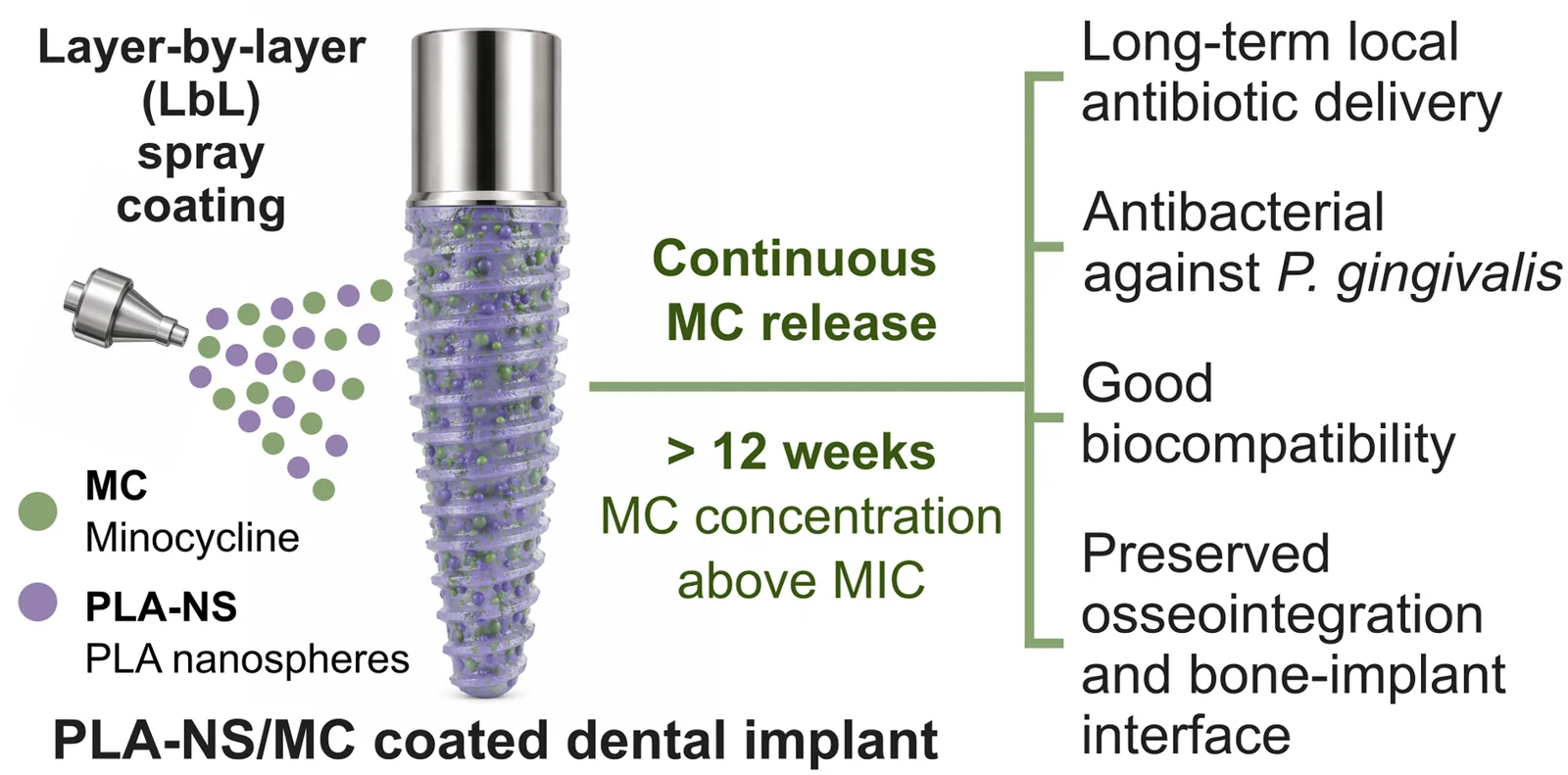

## 1 Introduction

Dental implants are among the most widely used implantable biomaterials for the replacement of missing teeth due to their excellent mechanical properties, biocompatibility, and ability to achieve long-term osseointegration.^1,2^ Despite their high clinical success rates, implant-associated infections remain a major challenge that can compromise tissue integration and long-term implant performance.^3,4^ The success of a dental implant depends not only on its ability to establish stable bone-to-implant contact but also on its resistance to bacterial colonization during the healing and functional phases.

Peri-implantitis is a biofilm-mediated inflammatory disease characterized by bacterial colonization of implant surfaces, inflammation of the surrounding soft tissues, and progressive loss of supporting bone.^3^ The disease is associated with a microbial community dominated by anaerobic Gram-negative pathogens, including *Porphyromonas gingivalis*, *Tannerella forsythia*, and *Fusobacterium nucleatum*.^5^ Among these microorganisms, *P. gingivalis* is considered a major periodontal pathogen because of its ability to modulate host immune responses, disrupt tissue homeostasis, and promote peri-implant bone destruction.^6^ Furthermore, *P. gingivalis* contributes to the development and maturation of polymicrobial biofilms on implant surfaces, where the extracellular matrix provides a protective environment that limits the penetration of antimicrobial agents and shields bacteria from host immune defenses.^7^ Once established, these biofilms are difficult to eradicate and may ultimately result in implant failure.

To reduce bacterial colonization and the risk of peri-implant infection, various implant surface modification strategies have been developed.^8,9^ Antibiotic-loaded coatings have attracted considerable interest because they provide localized antimicrobial activity directly at the implant-tissue interface while minimizing systemic exposure. Antimicrobial agents such as minocycline (MC),^10,11^ gentamicin,^12^ and vancomycin^12^ have been widely used to treat bacterial infections and inhibit bacterial proliferation. Among these agents, MC, a semisynthetic tetracycline antibiotic, exhibits broad-spectrum antibacterial activity against periodontal pathogens, including *P. gingivalis*.^13^ In addition to its antimicrobial activity, MC has been shown to reduce bone resorption, limit soft tissue destruction, and promote bone regeneration, making it an attractive therapeutic agent for dental implant applications.^14,15^ Despite these advantages, previously reported MC-coated implants exhibit limited drug loading and release the antibiotic for only a short period, typically up to 7 days, which may be insufficient to prevent bacterial colonization throughout the early stages of implant healing.^11,16^ Therefore, there remains a need for implant coatings capable of providing prolonged local antibiotic release while maintaining a simple and scalable fabrication process.

Poly(d,l-lactic acid) (PLA) has been extensively investigated as a biodegradable coating material because of its excellent biocompatibility, biodegradability, and approval for biomedical applications.^17,18^ PLA-based coatings have been successfully applied to titanium orthopedic and dental implants as carriers for antibiotics and other bioactive molecules while preserving the mechanical integrity of the metallic substrate.^19,20^ Most PLA-based drug-eluting coatings rely on encapsulating therapeutic agents within polymeric particles or films prior to coating. While these systems can prolong drug release, they often require additional formulation and encapsulation steps that increase fabrication complexity and may limit drug loading efficiency. Additionally, layer-by-layer (LbL) coating has emerged as a versatile surface engineering strategy for fabricating multifunctional implant coatings through the sequential deposition of functional layers.^21,22^ More recently, LbL coatings have been extended to the co-deposition of free therapeutic agents and polymeric nanoparticles to improve localized drug delivery and provide sustained release from implant surfaces.^23^

Herein, we report a PLA nanosphere (PLA-NS)/MC LbL coating for titanium dental implants. Drug-free PLA-NS and free MC were co-deposited to form the multilayer coating, followed by a PLA-NS capping layer to regulate drug release. This coating strategy enables long-term local antibiotic delivery while maintaining implant biocompatibility. The coating layers were fabricated using a combination of dip coating and automated spray coating, and the effects of coating architecture on drug release were systematically evaluated. The release of MC was evaluated under simulated oral conditions. The antibacterial performance of the coated implants against *P*. *gingivalis* was subsequently assessed using implant immersion and agar diffusion assays to determine both the antibacterial efficacy and the duration of antibacterial activity. The biological performance of the coating was further evaluated through *in vitro* hemocompatibility and cytocompatibility testing in accordance with ISO 10993 standards*. In vivo* biocompatibility was subsequently assessed through irritation, sensitization, and implantation studies, followed by histological evaluation of the bone-implant interface after 12 weeks in a rabbit femoral model. Together, these investigations provide a comprehensive assessment of the coating from material fabrication to biological performance. This work demonstrates that the PLA-NS-based LbL coating provides continuous local MC delivery, long-term antibacterial activity against *P. gingivalis*, and good biological compatibility without compromising osseointegration, supporting its potential as a drug-eluting coating platform for dental implants and other implantable metallic devices requiring localized therapeutic delivery.

## 2 Experimental section

### 2.1 Materials

Commercially pure titanium grade 4 dental implants (diameter: 5 mm; length: 14 mm) with sandblasted and acid-etched surfaces were used in this study. Minocycline hydrochloride (MC) was purchased from Shandong Deshang Chemical Co., Ltd (China). Poly(d,l-lactic acid) (PLA; molecular weight 70 kDa) was obtained from NatureWorks LLC (USA). Dichloromethane (DCM) and disodium hydrogen phosphate (Na_2_HPO_4_) were purchased from RCI Labscan Ltd (Thailand). Sodium chloride (NaCl), potassium dihydrogen phosphate (KH_2_PO_4_), and potassium chloride (KCl) were obtained from EMSURE® and Univar Solutions LLC, respectively. *Porphyromonas gingivalis* ATCC 33277 was obtained from the American Type Culture Collection (ATCC, Manassas, VA, USA). Automated dip- and spray-coating equipment used for implant coating were developed by IET SOLUTION Co., Ltd (Thailand). Minimum Essential Medium (MEM), fetal bovine serum (FBS) and penicillin-streptomycin (Pen-Strep) were obtained from Gibco (USA), and MTT was purchased from Invitrogen (USA). All chemicals were used as received unless otherwise specified.

### 2.2 PLA-NS fabrication

PLA-NS were prepared using a single oil-in-water emulsion solvent evaporation method. Briefly, PLA was dissolved in DCM to form the organic phase, while Pluronic F-127 was dissolved in deionized (DI) water to prepare the aqueous phase. The PLA solution was gradually added to the aqueous phase under stirring to form an emulsion, which was subsequently sonicated using a probe sonicator (Sonics Vibra-Cell™ CV18) to generate nanospheres. The resulting emulsion was stirred overnight at room temperature to allow complete evaporation of the organic solvent. The PLA-NS suspension was then centrifuged at 18 000 rpm and 4 °C for 40 min (Sorvall LYNX 6000 Centrifuge) to remove excess surfactant. The collected pellets were washed and re-dispersed in DI water, followed by filtration through a 0.45 µm membrane filter to remove aggregates and impurities. The purified PLA-NS suspension was stored at 4 °C until further use.

The particle size and zeta potential of the PLA-NS were determined using a nanoparticle analyzer (nanoPartica SZ-100V2, Horiba, Japan). All measurements were performed in triplicate. The morphology of the PLA-NS was further examined using field-emission scanning electron microscopy (FESEM; JSM-7610F PLUS, JEOL, Japan) operated at an accelerating voltage of 1.0 kV to evaluate the size, shape, and surface characteristics of the nanospheres.

### 2.3 LbL coating of dental implants

The dental implants were cleaned and immersed in sodium hydroxide (NaOH) solution at 60 °C overnight to modify the implant surface prior to coating. A 2% (w/v) PLA solution in DCM was first applied by dip coating to form the base layer. The implants were subsequently coated using a spray-based LbL technique. A coating suspension containing 1.5% (w/v) MC and 1.2% (w/v) PLA-NS was deposited onto the implant surface using an automated spray-coating system. The implants were subjected to 30, 60, 90, or 120 spray-coating cycles. During coating, the spray nozzle moved at a speed of 1.0 cm s^−1^ while the implant rotated at 100 rpm to ensure uniform coating deposition. A drying period of 1 min was incorporated after each spray cycle. Following completion of the PLA-NS/MC coating, a final PLA-NS layer was applied for 100 spray cycles. The coated implants were then annealed at 45 °C in a controlled climatic chamber, sterilized by gamma irradiation at a dose of 25 kGy, and packaged in sealed bags until further characterization and evaluation.

The surface morphology of the uncoated and coated dental implants was examined using scanning electron microscopy (SEM; JSM-IT500LA, JEOL, Japan). Prior to imaging, the implant specimens were sputter-coated with platinum for 10 min to enhance surface conductivity. The surface topography and coating morphology were then observed using SEM operated at an accelerating voltage of 5 kV.

### 2.4 *In vitro* drug release study

MC-coated dental implants were immersed in artificial saliva (AS; pH 6.8) to evaluate the release profile of MC under simulated oral conditions. Each implant was placed in 1.3 mL of AS and incubated at 37 °C in an orbital shaker operating at 90 rpm. To evaluate the initial burst release, the release medium was collected and replaced with fresh AS after 3, 6, and 12 h. Subsequently, samples were collected and replaced every 2 days until Day 28, followed by weekly medium replacement until the end of the study period to assess long-term drug release. The collected release media were analyzed using a UV-Vis spectrophotometer at a wavelength of 352 nm to determine the concentration of released MC.

### 2.5 *In vitro* cytotoxicity assessment

The *in vitro* cytotoxicity of the uncoated and MC-coated dental implants was evaluated in accordance with ISO 10993-5:2009 and ISO 10993-12:2021. Zinc acetate was used as the control material, with 4 ppm serving as the negative control (NC) and 18 ppm as the positive control (PC). Implant extracts were first prepared in complete cell culture medium according to the extraction conditions specified in ISO 10993-12:2021. L929 fibroblast cells were seeded into 96-well plates at a density of 1 × 10^4^ cells per well in MEM supplemented with 10% (v/v) FBS and 1% (v/v) Pen-Strep incubated at 37 °C and 5% CO_2_ for 24 h. The culture medium was then replaced with the implant extracts, and the cells were further incubated for 24 h. Subsequently, the culture medium was replaced with a serum-free, phenol red-free MTT medium, and the cells were incubated for 2 h. The MTT solution was removed, and isopropanol was added to dissolve the resulting formazan crystals. Absorbance was measured at 570 nm using a microplate reader, and cell viability was determined relative to untreated L929 cells cultured in complete MEM.

### 2.6 *In vitro* hemolytic evaluation

The hemolytic potential of the uncoated and MC-coated dental implants was evaluated in accordance with ASTM F756-17. Implant extracts were prepared and incubated with rabbit red blood cells (RBCs) in magnesium- and calcium-free PBS. High-density polyethylene (HDPE) sheet and polyvinyl chloride (PVC) sheet were used as the negative and positive controls, respectively. The mixtures were incubated at 37 °C for 3 h with periodic inversion to ensure adequate contact between the extracts and RBCs. Following incubation, the samples were centrifuged, and the released hemoglobin in the supernatant was quantified using Drabkin's reagent and a UV-Vis spectrophotometer at 540 nm. The percentage hemolysis was subsequently calculated relative to the positive and negative controls.

### 2.7 *In vitro* antibacterial activity by implant immersion assay

The antibacterial activity of the uncoated and MC-coated dental implants against *P. gingivalis* ATCC 33277 was evaluated using an implant immersion assay. *P. gingivalis* was initially cultured on 5% blood agar plates under anaerobic conditions at 37 °C for 5 days. Bacterial colonies were then transferred to anaerobic blood broth (ABB) and incubated for 5 days. The bacterial suspension was adjusted to approximately 3 × 10^8^ CFU mL^−1^ using McFarland standard No. 1 and subsequently diluted to 1 × 10^6^ CFU mL^−1^ as the working inoculum. For the antibacterial assay, 100 µL of the bacterial inoculum was mixed with 900 µL of ABB to obtain a final volume of 1 mL. The uncoated and MC-coated dental implants were immersed in the bacterial suspension and incubated anaerobically at 37 °C for 7 days. After incubation, the bacterial suspensions were serially diluted and plated onto 5% blood agar plates, followed by anaerobic incubation at 37 °C for 5 days. The number of viable bacteria was determined using the colony counting technique and expressed as CFU mL^−1^.

### 2.8 *In vitro* antibacterial activity by agar diffusion assay

The long-term antibacterial activity of the MC-coated dental implants was evaluated using an agar diffusion assay against *P. gingivalis* ATCC 33277. Bacterial cultures were grown anaerobically at 37 °C on 5% blood agar plates and subsequently suspended in anaerobic blood broth. The bacterial suspension was adjusted to approximately 3 × 10^8^ CFU mL^−1^ using McFarland standard No. 1 and uniformly spread onto fresh 5% blood agar plates. The MC-coated dental implants were then aseptically placed on the inoculated agar surface and incubated anaerobically at 37 °C. Antibacterial activity was assessed by measuring the diameter of the inhibition zone surrounding each implant after 3 days of incubation. To evaluate the duration of antibacterial activity, the implants were transferred to freshly inoculated agar plates at predetermined intervals, and the inhibition zone was measured at each time point.

### 2.9 Accelerated shelf-life evaluation

Accelerated shelf-life testing was performed to evaluate the storage stability of the MC-coated dental implants. The coated implants were packaged according to an ISO 13485-compliant packaging procedure and subjected to accelerated aging at 50 °C for 91 days, corresponding to an estimated shelf-life of 1 year at 30 °C, based on the accelerated aging calculation described in ASTM F1980-22.

A total of six MC-coated dental implants were evaluated, with three freshly prepared implants assigned to the Fresh group and three accelerated-aged implants assigned to the 1 Year (Accelerated) group. The antibacterial activity of both groups against *P. gingivalis* was evaluated using the agar diffusion assay. The implants were transferred to fresh agar plates at 7-day intervals to assess the duration of antibacterial activity over 105 days. The diameter of the inhibition zone was measured at each time point and compared between the Fresh and 1 Year (Accelerated) groups.

### 2.10 *In vivo* intracutaneous irritation test

The irritation potential of the MC-coated dental implants was evaluated using an intracutaneous reactivity test in New Zealand White rabbits in accordance with ISO 10993-23:2021. Implant extracts were prepared following ISO 10993-12:2021 using polar (0.9% sodium chloride injection) and non-polar (sesame oil) extraction vehicles. Briefly, the coated dental implants were extracted at a ratio of 0.2 g mL^−1^ in the respective extraction vehicles and incubated at 50 ± 2 °C for 72 ± 2 h under continuous agitation at 100 rpm. Corresponding vehicle controls consisting of 0.9% sodium chloride injection and sesame oil without the test item were prepared under identical conditions.

Three female New Zealand White rabbits were acclimatized for 5 days prior to the study. Approximately 24 h before administration, the fur on both sides of the dorsal flank region was clipped and the injection sites were marked. Each rabbit received intracutaneous injections of the polar and non-polar vehicle controls on the right side of the spinal column and the corresponding polar and non-polar implant extracts on the left side. A volume of 0.2 mL was injected at five separate sites for each extract and control.

The injection sites were examined for erythema and edema at 24, 48, and 72 h after administration. Body weights and general clinical observations were also recorded throughout the study period. The irritation response was evaluated according to the scoring criteria specified in ISO 10993-23:2021.

### 2.11 *In vivo* skin sensitization test

The sensitization potential of the MC-coated dental implants was evaluated using the Guinea Pig Maximization Test (GPMT) in accordance with ISO 10993-10:2021. Implant extracts were prepared according to ISO 10993-12:2021 using polar (0.9% sodium chloride) and non-polar (sesame oil) extraction vehicles. Thirty female guinea pigs were randomly assigned to four groups: polar vehicle control, polar extract, non-polar vehicle control, and non-polar extract. Prior to treatment, the fur at the application sites was clipped without damaging the skin.

On Day 1, intradermal induction was performed in the shoulder region using Freund's complete adjuvant (FCA), vehicle controls, or implant extracts according to the GPMT protocol. Skin reactions were evaluated 24 and 48 h after injection using the Draize scoring system. On Day 8, topical induction was carried out by applying filter paper patches (2 cm × 4 cm) saturated with 0.5 mL of the respective vehicle control or implant extract to the clipped dorsal skin. The patches were secured with a semi-occlusive dressing and maintained in contact with the skin for 48 h. Skin responses were evaluated after patch removal.

Following a 14 day induction period, challenge exposure was performed on Day 22. Fresh filter paper patches containing 0.5 mL of the corresponding vehicle control or implant extract were applied to separate sites on the animals and maintained under semi-occlusive dressing for 24 h. Skin reactions were assessed at 24 and 48 h after patch removal using the Magnusson and Kligman grading scale. Animals were monitored daily for clinical signs, body weight changes, and general health status, and were examined for mortality and morbidity throughout the study period.

### 2.12 *In vivo* implantation study

The *in vivo* biocompatibility of the dental implants was evaluated in accordance with ISO 10993-6:2016. All animal experiments were approved by the Institutional Animal Care and Use Committee (IACUC) of the National Laboratory Animal Center, Mahidol University (Approval No. RA2022-33) and conducted in accordance with applicable guidelines and regulations for the care and use of laboratory animals. Ten male New Zealand White rabbits, approximately 4 months of age and weighing 3502.7 ± 159.0 g, were used in the study. Each rabbit received an uncoated dental implant in the left femoral bone (control group) and an MC-coated dental implant in the right femoral bone (test group). Animals were acclimatized prior to implantation and monitored for general health throughout the study.

Anesthesia was induced by intramuscular administration of xylazine (5 mg per kg) and maintained with 2–4% isoflurane inhalation. Following shaving and aseptic preparation of the surgical site, a skin incision was made to expose the distal femoral bone. Implant sites were prepared using a surgical drill under saline irrigation, and dental implants (5 mm diameter × 14 mm length) were inserted into the prepared defects. The surgical wounds were closed using absorbable sutures. Postoperative analgesia consisted of morphine (1–2 mg kg^−1^, subcutaneous) for the first 2 days, followed by tramadol (5 mg kg^−1^, subcutaneous) from Days 3–5. Enrofloxacin (5–10 mg kg^−1^, subcutaneous) was administered for 5 days following surgery. Animals were monitored daily for clinical signs, body weight changes, feed and water consumption, and wound healing.

After 12 weeks of implantation, the animals were euthanized by intravenous overdose of thiopental sodium. Implantation sites were collected and fixed in 10% neutral buffered formalin. Following fixation, the specimens were decalcified using EDTA-based decalcifying solution, processed routinely, embedded in paraffin, sectioned at 8 µm thickness, and stained with hematoxylin and eosin (H&E). Histological evaluation was performed using a light microscope (Leica DM2000 LED, Germany) equipped with Leica Application Suite software (version 4.9.0). Tissue responses surrounding the implants were assessed according to ISO 10993-6:2016 and INHAND guidelines, including inflammatory cell infiltration, foreign body reaction, necrosis, fibrosis, neovascularization, and bone healing at the implant–tissue interface.

## 3 Results and discussion

### 3.1 Fabrication and characterization of MC-coated dental implants

The MC-coated dental implants were fabricated using a three-step coating process (Fig. 1A). First, a thin PLA primer layer was applied by dip coating using a PLA solution in DCM to improve coating adhesion. This was followed by LbL spray coating with a suspension containing drug-free PLA-NS and free MC, which were co-deposited onto the implant surface to construct the multilayer coating. Finally, a PLA-NS-only capping layer was deposited for an additional 100 spray cycles to further regulate MC release and protect the underlying multilayer structure.

**Fig. 1:**
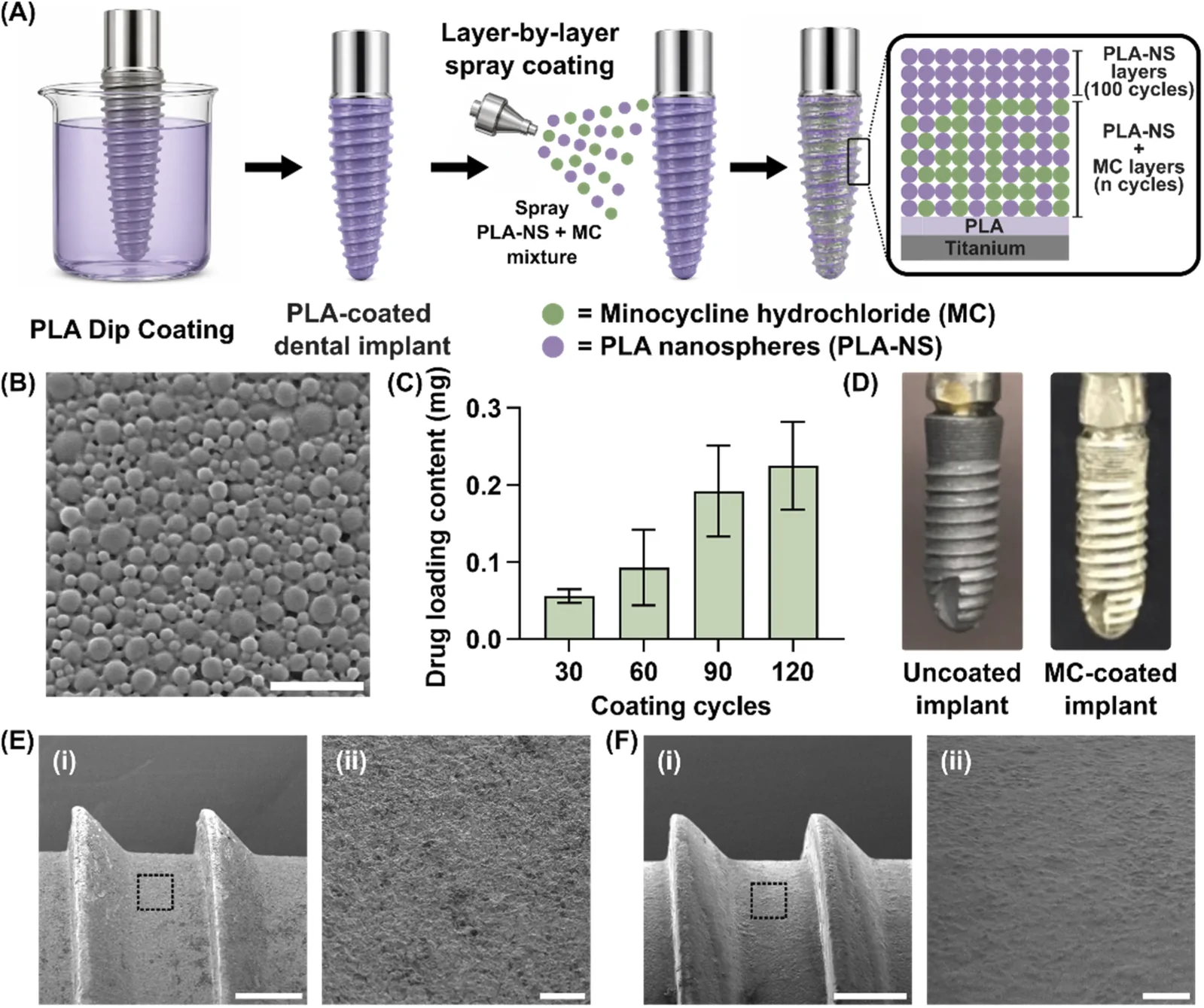
Fabrication and characterization of MC-coated dental implants. (A) Schematic illustration of the MC-coated dental implant fabrication process. (B) Representative FESEM image of PLA-NS. Scale bars: 1 µm. (C) Average drug loading content of MC-coated dental implants with different coating layers. Data presented as mean ± SD (*n* = 3, technical replicates). (D) Photographs of the uncoated and MC-coated dental implants. The MC-coated implant was prepared using 120 PLA-NS/MC co-deposition cycles followed by 100 PLA-NS capping cycles. Representative SEM images of (E) the uncoated dental implant and (F) the MC-coated dental implant after the complete coating process. For panels (E and F), (i) shows the low-magnification image, and (ii) shows the high-magnification image of the threaded region. Scale bars: (i) 500 µm; (ii) 5 µm.

PLA-NS were initially prepared using a single-emulsion solvent evaporation method. FESEM confirmed their spherical morphology, with an average diameter of 182.2 ± 45.5 nm (Fig. 1B). DLS analysis of the PLA-NS dispersion showed an intensity-based hydrodynamic diameter of 179.3 ± 0.2 nm and a polydispersity index (PDI) of 0.15, indicating a relatively narrow size distribution. The slight discrepancy between FESEM and DLS measurements can be attributed to the different measurement principles. FESEM determines the geometric size of dried particles under vacuum conditions, where sample dehydration may induce shrinkage, flattening, or particle aggregation. In contrast, DLS measures the hydrodynamic diameter of particles in suspension. Typically, nanoparticles in the 100–200 nm range have been reported to be suitable for coating biomedical surfaces because their relatively small size can facilitate surface coverage and conformal deposition.^24^ In line with previous reports, nanoparticle-based LbL coatings have been shown to enhance coating adhesion and homogeneity on medical devices.^25,26^

The surface charge of the PLA-NS was evaluated by zeta potential measurements. The nanospheres exhibited a negative zeta potential with an average of −55.60 ± 0.30 mV. This negative value confirms the presence of terminal carboxylic groups in PLA and indicates good colloidal stability due to electrostatic repulsion between particles.^27^ Moreover, the negatively charged surface may contribute to biological performance by repelling the similarly negatively charged bacterial cell walls, thereby reducing microbial adhesion and potentially lowering the risk of biofilm formation.^28^

Following fabrication of the PLA-NS, the dental implants were initially dip-coated in a 2% (w/v) PLA solution in DCM to form a thin polymer primer layer on the implant surface. This primer layer improved the adhesion and stability of the subsequently deposited coating while providing conformal coverage of the implant surface.^29^ The implants were then spray-coated using an automated LbL system with a suspension containing 1.5% (w/v) MC and 1.2% (w/v) drug-free PLA-NS to construct the multilayer coating. Finally, an outermost capping layer of PLA-NS was deposited with an additional 100 spray cycles to regulate MC release and protect the underlying multilayer coating. To investigate the effect of coating buildup on drug loading, implants were fabricated using 30, 60, 90, or 120 PLA-NS/MC spray-coating cycles, followed by a 100-cycle PLA-NS capping layer. Drug loading was quantified to investigate the influence of spray cycle number on MC incorporation within the coating layer. The results showed that the amount of loaded drug increased progressively with increasing spray cycles, demonstrating successful accumulation of the PLA-NS/MC layers during the LbL deposition process (Fig. 1C). Drug loading for the 30, 60, 90, and 120 spray cycles was measured at 0.056 ± 0.009, 0.093 ± 0.049, 0.192 ± 0.059, and 0.225 ± 0.057 mg, respectively. This trend is consistent with previous studies reporting that increasing the number of LbL deposition cycles enhances coating buildup and drug incorporation efficiency.^26^ Compared with conventional single-layer coatings, the multilayer architecture provides several advantages.^30,31^ The repeated co-deposition of PLA-NS and MC increased drug loading while creating a more tortuous diffusion pathway for drug transport. In addition, the outermost 100-cycle PLA-NS capping layer provides an additional diffusion barrier that may further moderate MC release from the underlying multilayer coating. Collectively, these structural features contribute to prolonged local antibiotic release while maintaining a simple coating strategy without nanoparticle drug encapsulation. Because the 120 PLA-NS/MC deposition cycles, followed by the 100-cycle PLA-NS capping layer, yielded the highest drug loading, this coating configuration was selected for all subsequent drug release, antibacterial, and biocompatibility evaluations (Fig. 1D).

SEM analysis was performed to evaluate the surface morphology of the uncoated and MC-coated dental implants after 120 PLA-NS/MC deposition cycles followed by a 100-cycle PLA-NS capping layer (Fig. 1E and F). Both the low- and high-magnification SEM images were acquired from the threaded region of the implant, which represents the primary bone-contacting surface and the principal site for local antibiotic delivery. The low-magnification images provide an overview of the coated threaded surface, whereas the high-magnification images reveal the surface morphology in greater detail. The uncoated implant exhibited a relatively rough and irregular surface morphology, which may facilitate bacterial attachment and subsequent biofilm formation.^32^ In contrast, the coated implant displayed a smoother and more continuous surface morphology, indicating successful deposition of the PLA-NS/MC multilayer coating. Although coating uniformity in the grooves and implant neck was not quantitatively evaluated, the SEM observations confirm successful coating formation on the clinically relevant threaded implant surface.

### 3.2 *In vitro* drug release studies of MC-coated dental implants

The *in vitro* release study was conducted to further evaluate the release behavior of MC from the coated dental implants. The release profile was assessed in AS to better simulate the oral environment and evaluate the performance of the coating under physiological conditions. The cumulative release profile exhibited a biphasic release pattern consisting of an initial burst release followed by a slower continuous release phase. An initial rapid release of MC was observed within the first hour (Fig. 2A), followed by a continuous release of MC for more than 90 days (Fig. 2B). This initial burst release phase was attributed to MC molecules located at or near the coating surface, which were readily exposed to the release medium and therefore diffused rapidly into the surrounding environment. In addition, MC is a highly water-soluble drug, which facilitates rapid dissolution upon contact with the aqueous medium.^13^ Similar burst-release behavior has been reported for other MC-coated implant systems, where the rapid release of surface-accessible drug provides early protection against implant-associated bacterial colonization and infection.^33–35^ This initial burst release is particularly advantageous for dental implants, as it can inhibit the immediate attachment and colonization of bacteria on the implant surface following implantation, thereby reducing the risk of early biofilm formation and peri-implant infection.^10,36^ Moreover, the MC concentration released during the initial burst phase remained within a biocompatible range and has been reported not to induce significant necrosis or apoptosis in oral epithelial cells, supporting its suitability for local antimicrobial delivery in dental applications.^37^

**Fig. 2:**
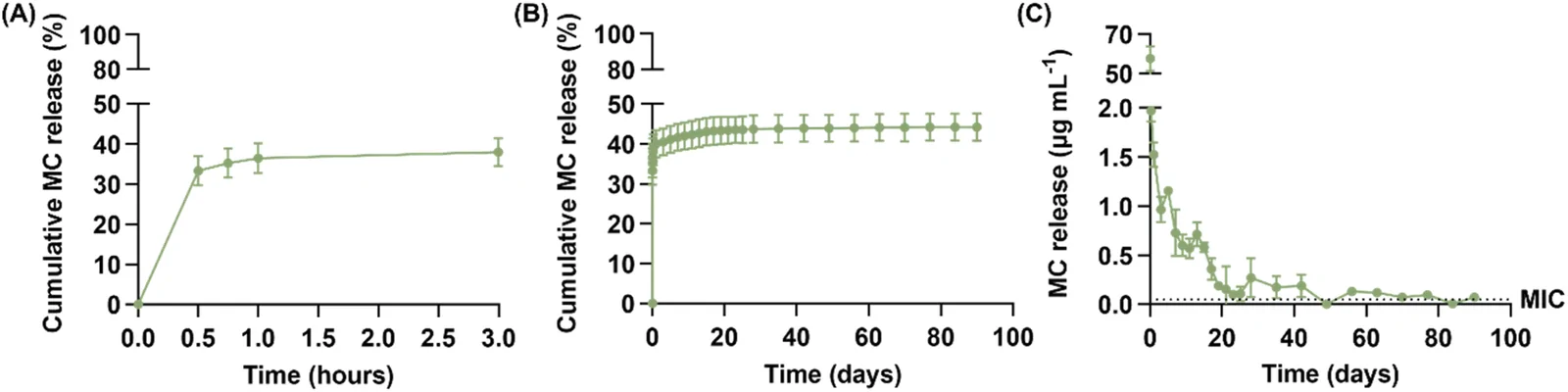
*In vitro* drug release studies of MC-coated dental implants. (A) Cumulative MC release during the first 3 h. (B) Cumulative MC release over 90 days in AS. (C) MC concentration released at each sampling interval during the 90-day release study. The dashed line indicates the MIC of MC against *P. gingivalis*. Data presented as mean ± SD (*n* = 3).

Following the initial burst release, a slower release phase was observed for up to 90 days (Fig. 2C). The average concentration of MC released during the first 15 days was 0.85 ± 0.32 µg mL^−1^, decreasing to 0.14 ± 0.09 µg mL^−1^ from day 15 to day 90. The reduction in release rate is likely attributed to depletion of accessible drug molecules and the increasing diffusion distance from the inner layers of the coating. Notably, the average released MC concentration during both periods remained well above the MIC against *P. gingivalis* (0.03 µg mL^−1^) and some other strains associated with periodontitis.^38^ These results indicate that the coating was capable of maintaining antibacterial concentrations throughout the 90-day release study. Previous studies on local MC delivery platforms for periodontitis treatment have demonstrated sustained drug release for approximately 11 weeks from MC-loaded depots,^39^ whereas microneedle patches typically provide sustained release for only 1–2 weeks.^40,41^ Likewise, MC-coated dental implants developed for peri-implantitis treatment have generally exhibited sustained drug release for only up to 7 days.^11^

To better understand the release mechanism of MC from the coated dental implants, several kinetic models were fitted to the release profile. Among the kinetic models evaluated, the Korsmeyer-Peppas model provided the highest goodness-of-fit (*R*^2^ = 0.877), compared with the zero-order (*R*^2^ = 0.157) and first-order (*R*^2^ = 0.055) models (Table 1). Although the model exhibited only a moderate fit, it described the experimental data better than the other models, suggesting that diffusion contributed substantially to MC release. The release profile exhibited an initial burst release followed by a prolonged sustained release, indicating that the overall release behavior cannot be fully represented by a single kinetic model. Nevertheless, the release exponent (*n*) was below 0.45, indicating a Fickian diffusion mechanism during the diffusion-controlled stage.^42,43^ Under this mechanism, drug transport occurs primarily through the concentration gradient between the multilayer coating and the surrounding release medium. The moderate goodness-of-fit is likely attributable to the biphasic release profile, which combines an initial burst release with sustained diffusion through the multilayer PLA-NS coating.

**Table 1:** The release kinetic analysis of the release behavior of MC from dental implants

Zero-order model		First-order model		Korsmeyer-Peppas model		
*K*_0_	*R* ^2^	*K*_1_	*R* ^2^	*n*	*K*_2_	*R* ^2^
0.263	0.157	0.007	0.055	0.028	0.897	0.877

### 3.3 *In vitro* antibacterial activity of MC-coated dental implants

To verify that the released MC remained biologically active after release from the coating, its antibacterial activity was first evaluated using an implant immersion assay followed by a bacterial colony assay. In this study, *P. gingivalis* was selected as the test microorganism because it is a major periodontal pathogen associated with peri-implantitis and plays a significant role in the initiation and progression of peri-implant infections.^44,45^ Uncoated and MC-coated dental implants were immersed in *P. gingivalis* suspensions and incubated for 7 days under anaerobic conditions prior to bacterial quantification. After incubation for 7 days, the uncoated implant group exhibited substantial bacterial growth, reaching approximately 8.52 × 10^5^ CFU mL^−1^, whereas no detectable bacterial colonies were observed in the coated implant group (Fig. 3A). This complete inhibition of bacterial growth is consistent with the elevated MC concentrations released during the initial phase of the release profile. The ability of the released MC to suppress planktonic *P. gingivalis* cells is particularly important during the early stages following implantation, as these free-floating bacteria are responsible for the initial attachment and subsequent formation of biofilms on implant surfaces.^7^

**Fig. 3:**
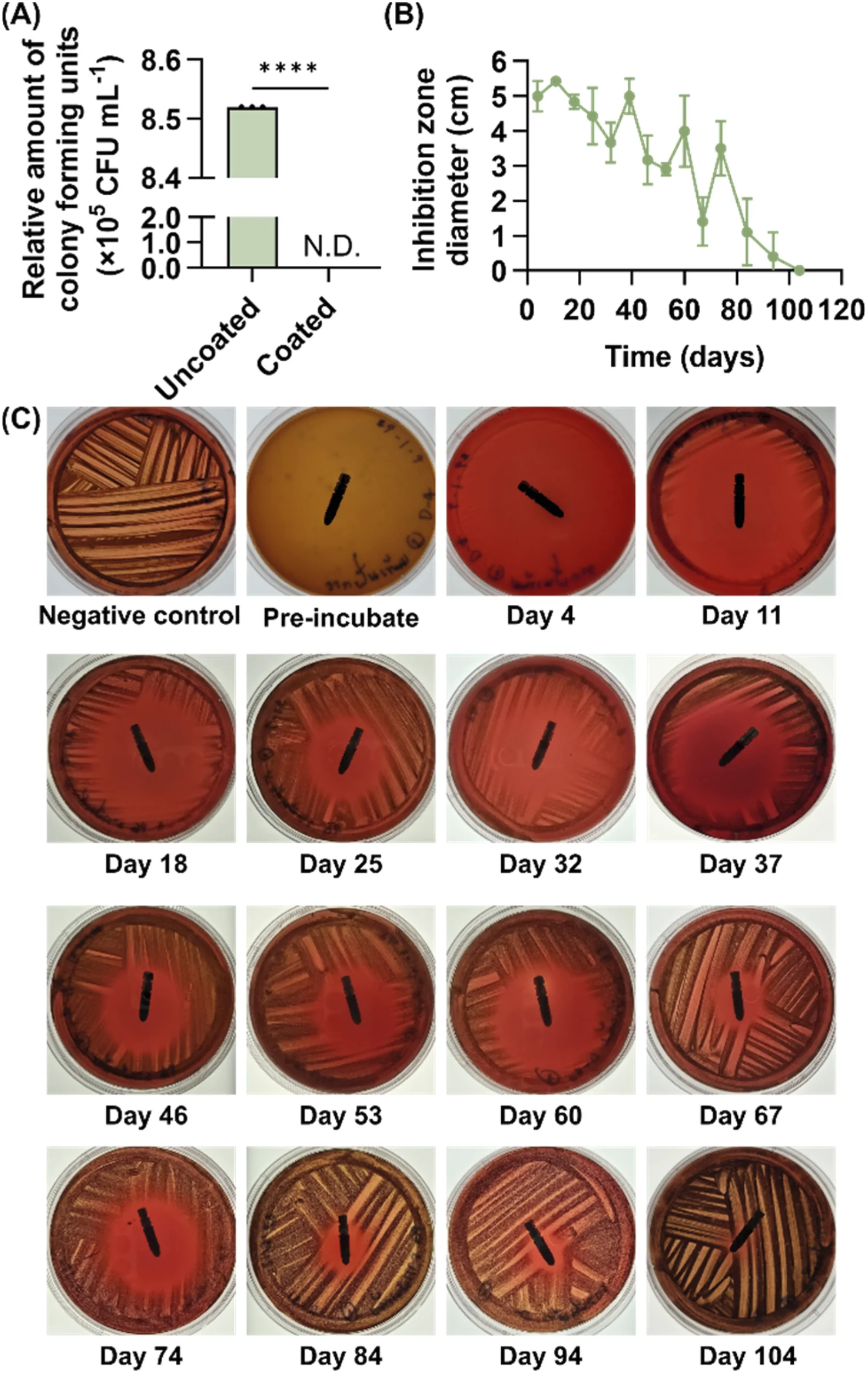
*In vitro* antibacterial activity of MC-coated dental implants. (A) Viable *P. gingivalis* concentrations (CFU mL^−1^) after 7 days of incubation with uncoated and MC-coated dental implants. Values represent mean ± SD. N.D. indicates no detectable bacterial growth. Statistical analysis was performed using an unpaired *t*-test (****p < 0.0001). (B) Diameters of the inhibition zone against *P. gingivalis* produced by MC-coated dental implants over the study period. Values are presented as mean ± SD. (C) Representative agar plate images showing the zones of inhibition surrounding MC-coated dental implants against *P. gingivalis* at different time intervals during the antibacterial activity study.

Given the release of MC at concentrations above the MIC against *P. gingivalis* for over 90 days, the antibacterial activity of the released drug was further evaluated to determine whether the coating could effectively inhibit bacterial growth over the release period. The antibacterial activity of the coated dental implants was evaluated using an agar diffusion assay by measuring the diameter of the zone of inhibition surrounding the implants (Fig. 3B and C). The MC-coated dental implants were transferred to freshly inoculated *P. gingivalis* agar plates after each measurement interval, and the zone of inhibition was subsequently determined. This process was repeated throughout the study period to evaluate the antibacterial activity of the coating. The results demonstrated that the MC-coated dental implants retained antibacterial activity against *P. gingivalis* for up to 94 days. However, no detectable inhibition zone was observed on day 104, suggesting that the remaining MC release had fallen below the concentration required to suppress bacterial growth. This finding is consistent with the gradual decline in drug release observed during the later stages of the release study. The presence of an inhibition zone for up to 94 days indicates that the released MC remained biologically active throughout most of the study period and was able to diffuse from the coating into the surrounding medium to suppress bacterial proliferation. Maintaining antibacterial activity for up to 94 days could potentially help reduce bacterial colonization at the implant–tissue interface and lower the risk of implant-associated infection.^46,47^ The ability of the PLA-NS coating to maintain antibacterial efficacy for approximately three months highlights its potential as a long-term local drug delivery platform for preventing peri-implant infections.

To further evaluate the storage stability of the coating, accelerated-aged implants (1 year equivalent) were assessed for antibacterial activity. Measurements were performed over 105 days at 7-day intervals. Comparable antibacterial performance was observed between the fresh and accelerated-aged groups, with no significant differences in the duration of inhibition zone formation throughout the study (Fig. 4). Both groups exhibited detectable inhibition zones for up to 91 days, after which the inhibition zones gradually disappeared. Similar to the earlier results, no detectable inhibition zone was observed after day 105 in either group, indicating that the released MC concentration had declined below the MIC required to suppress *P. gingivalis* growth. These findings demonstrate that accelerated aging, equivalent to 1 year of storage, did not adversely affect the antibacterial performance of the PLA-NS/MC coating, supporting its projected shelf-life stability under the evaluated storage conditions.

**Fig. 4:**
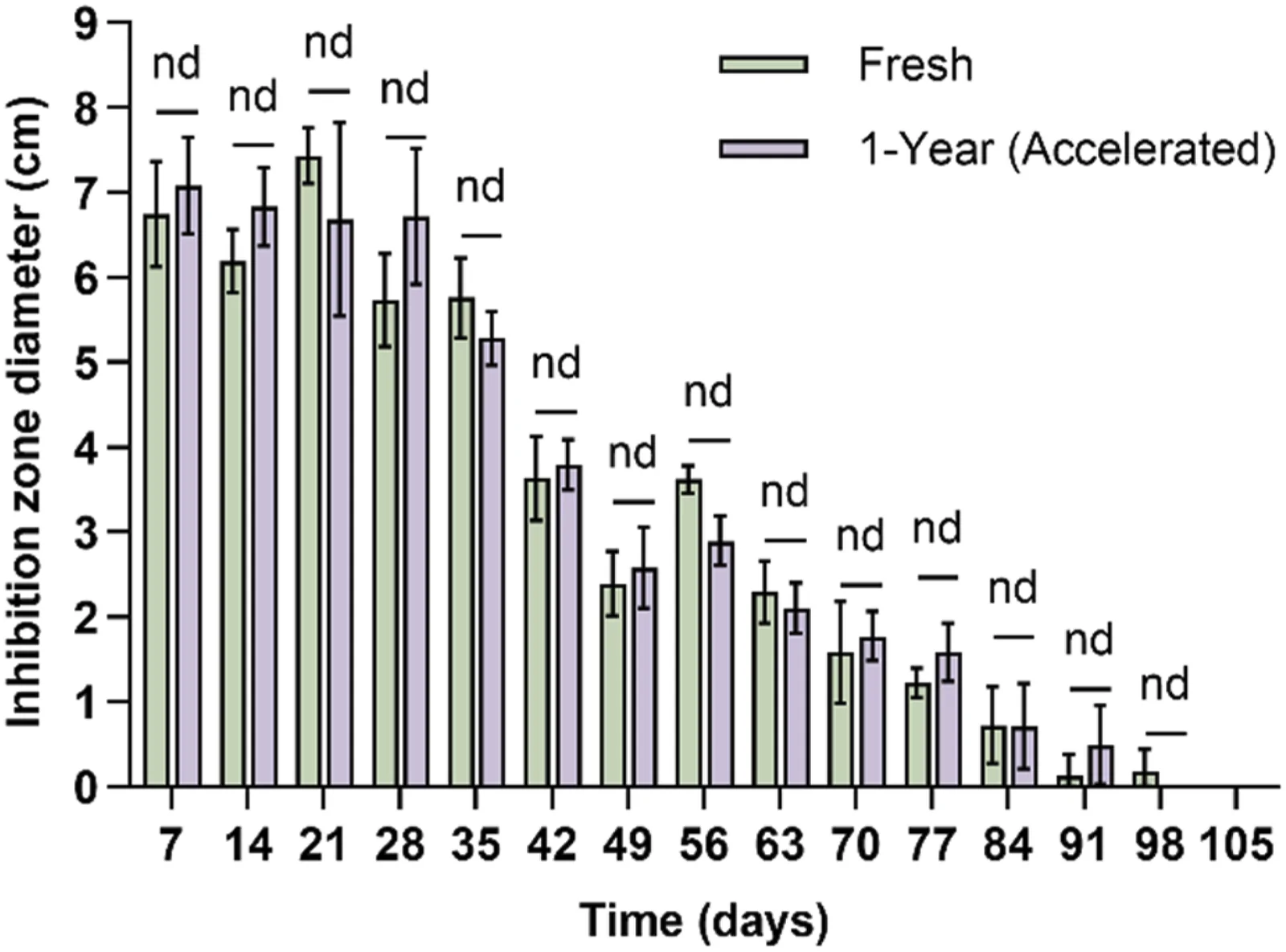
Accelerated shelf-life evaluation of MC-coated dental implants. Comparison of the duration of antibacterial activity between freshly prepared (Fresh) and accelerated-aged (1 Year equivalent) MC-coated dental implants against *P. gingivalis*. Accelerated aging was performed at 50 °C for 91 days, corresponding to an estimated shelf-life of 1 year at 30 °C. Data are presented as mean ± SD (*n* = 3). nd, no significant difference (*p* > 0.05).

### 3.4 *In vitro* biocompatibility studies of MC-coated dental implants

Having demonstrated the successful fabrication of MC-coated dental implants capable of sustained drug release and antibacterial activity for the prevention of peri-implant infections, the biocompatibility of the coating was subsequently evaluated. *In vitro* biocompatibility studies were performed through hemolysis testing using rabbit RBCs and cytotoxicity assessment against L929 fibroblast cells to determine the safety of the coating for potential dental implant applications prior to preclinical *in vivo* evaluation.

Since dental implants are blood-contacting implantable devices, hemocompatibility is an important consideration for their clinical application.^48^ The *in vitro* hemolysis assay was conducted using extracts obtained from the dental implants following incubation in PBS (Fig. 5A). The extracts were subsequently incubated with rabbit RBCs for 3 h, and the percentage of hemolysis was determined. The uncoated dental implants exhibited a hemolysis percentage of 0.13 ± 0.09%, while no detectable hemolysis was observed for the MC-coated dental implants. In comparison, the PVC sheet used as the positive reference material exhibited a hemolysis percentage of 4.43 ± 0.34%, whereas the HDPE sheet used as the negative reference material showed no detectable hemolysis. Although the uncoated implants exhibited a measurable hemolysis value, the percentage remained extremely low and well below the 5% threshold specified by ISO 10993-4 for non-hemolytic biomaterials. Therefore, both the uncoated and MC-coated dental implants can be classified as non-hemolytic materials, demonstrating hemocompatibility. These findings further indicate that the incorporation of MC and the PLA-NS coating did not adversely affect the hemocompatibility of the dental implants. Similar observations have been reported for PLA-coated metallic implants, where the application of a PLA coating reduced hemolytic activity compared with uncoated substrates, further supporting the hemocompatibility of PLA-based surface modifications.^49,50^

**Fig. 5:**
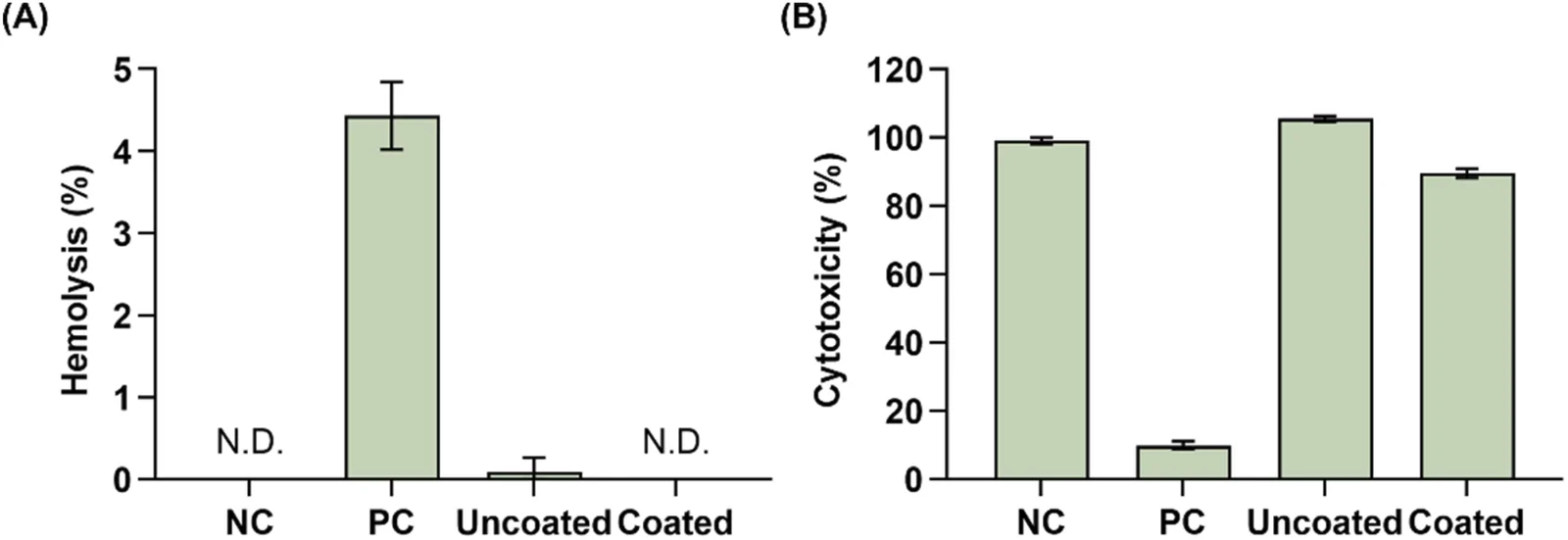
*In vitro* biocompatibility testing of MC-coated dental implants. (A) Hemolytic activity expressed as percentage hemolysis of rabbit red blood cells (RBCs). (B) Cytotoxicity assessed using L929 fibroblast cells. Data are presented as mean ± SD (*n* = 3). NC, negative control; PC, positive control; N.D., not detected.

The *in vitro* cytotoxicity assay was performed according to ISO 10993-5 to evaluate the biological safety of the uncoated and MC-coated dental implants. L929 fibroblast cells were incubated with extracts prepared from the implant specimens in complete cell culture medium, and cell viability was subsequently assessed to determine any cytotoxic effects associated with the coating system (Fig. 5B). As expected, the negative control exhibiting a cell viability of 99.1 ± 0.9%, while the positive control resulted in substantial cytotoxicity, with a viability of only 9.9 ± 1.1%. In comparison, L929 cells exposed to extracts from the uncoated and MC-coated dental implants exhibited viabilities of 105.5 ± 0.8% and 89.5 ± 1.4%, respectively. Although the MC-coated implants showed slightly lower cell viability than the uncoated implants, both values remained well above the 70% threshold specified by ISO 10993-5 for non-cytotoxic materials. Overall, the MC-coated dental implants exhibited *in vitro* biocompatibility, with no evidence of hemolytic or cytotoxic effects. The combination of favorable biological safety, sustained drug release, and long-term antibacterial activity supports the potential application of this coating system for the prevention of peri-implant infections.

### 3.5 *In vivo* biocompatibility evaluation of MC-coated dental implants

Having demonstrated *in vitro* hemocompatibility and cytocompatibility, the biocompatibility of the MC-coated dental implants was further evaluated through an *in vivo* intracutaneous irritation test in accordance with ISO 10993-23:2021. Implant extracts prepared using polar (0.9% sodium chloride injection) and non-polar (sesame oil) extraction vehicles were administered by intracutaneous injection. The injection sites were examined for signs of erythema and edema at 24, 48, and 72 h after administration. Throughout the study period, all animals remained clinically healthy, with no mortality, morbidity, or abnormal clinical signs observed. No erythema or edema was detected at any test or control injection site for either the polar or non-polar extracts at any observation time (Table 2). Based on the ISO 10993-23:2021 evaluation criteria, the MC-coated dental implant extracts were classified as non-irritating. These findings indicate that neither the coating materials nor the released MC induced adverse local tissue reactions, further supporting the biocompatibility of the developed coating system.

**Table 2:** Intracutaneous irritation scores of MC-coated dental implant extracts in rabbits according to ISO 10993-23:2021

Time	Polar vehicle (0.9% NaCl)		Polar extract		Non-polar vehicle (sesame oil)		Non-polar extract	
	ERY^a^	EDE^a^	ERY^a^	EDE^a^	ERY^a^	EDE^a^	ERY^a^	EDE^a^
24 h	0	0	0	0	0	0	0	0
48 h	0	0	0	0	0	0	0	0
72 h	0	0	0	0	0	0	0	0

a ERY = erythema; EDE = Edema.

To further evaluate the *in vivo* biological safety of the MC-coated dental implants, the sensitization potential of their polar and non-polar extracts was assessed in accordance with ISO 10993-10:2021. Guinea pigs were subjected to intradermal and topical induction with the vehicle controls or implant extracts, followed by challenge exposure on Day 22. Skin reactions were subsequently evaluated at 24 and 48 h after patch removal to assess delayed hypersensitivity responses. Throughout the study period, all animals remained clinically healthy, with no mortality, morbidity, or abnormal clinical signs observed. Following challenge exposure, no erythema, edema, or other cutaneous reactions were observed in any of the animals treated with either polar or non-polar extracts (Table 3). The skin responses were comparable to those of the corresponding vehicle control groups, and all animals received a Magnusson and Kligman score of 0. Consequently, the sensitization incidence was 0%, indicating that the MC-coated dental implant extracts did not induce delayed hypersensitivity reactions. The absence of sensitization responses is particularly important for implantable medical devices, as repeated or prolonged exposure to device materials and their leachable components may potentially trigger immune-mediated adverse reactions that could compromise long-term biocompatibility and clinical performance.

**Table 3:** Skin sensitization assessment of MC-coated dental implant extracts in guinea pigs according to ISO 10993-10:2021

Treatments	Magnusson and Kligman score		Sensitization incidence (%)^a^
	24 h	48 h	
Polar vehicle control	0	0	0
Polar extract	0	0	0
Non-polar vehicle control	0	0	0
Non-polar extracts	0	0	0

a Sensitization incidence (%) = number of animals with positive reaction/total animal × 100.

Following the evaluation of irritation and sensitization responses, the *in vivo* biocompatibility of the MC-coated dental implants was further investigated in a 12-week implantation study. Histological analysis was performed to investigate local tissue responses, inflammation, and bone-implant integration under long-term implantation conditions. Uncoated dental implants were implanted into the left femoral bone, whereas MC-coated dental implants were implanted into the right femoral bone (Fig. 6A). Throughout the study period, all animals remained clinically healthy, with no signs of abnormal behavior, lethargy, depression, wound complications, or other adverse clinical symptoms. Furthermore, no significant body weight loss was observed, and body weights increased steadily over the course of the study. Macroscopic examination revealed no visible lesions or abnormal tissue responses at the implantation sites. The surrounding soft tissues appeared healthy, with no evidence of inflammation, infection, or necrosis. Notably, bone remodeling was observed around the implantation sites, with newly formed bone covering most of the implant surfaces after 12 weeks.

**Fig. 6:**
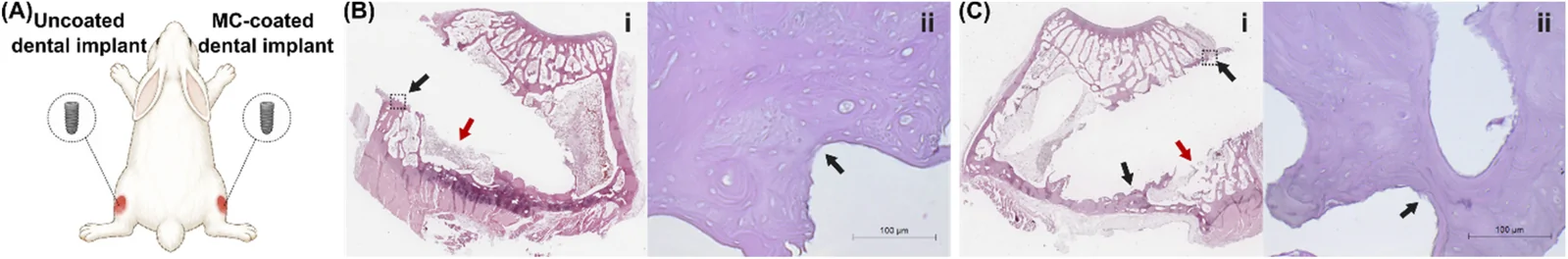
*In vivo* implantation assessment of MC-coated dental implants after 12 weeks. (A) Schematic illustration of the implantation study in New Zealand White rabbits, in which uncoated dental implants were implanted in the left femoral bone, and MC-coated dental implants were implanted in the right femoral bone. Representative H&E-stained histological sections of the implant–bone interface for (B) uncoated and (C) MC-coated dental implants. For panels (B and C), (i) represents the overall view of the implantation site and (ii) shows a higher-magnification image of the bone-implant interface. Black arrows indicate the bone-implant interface, and red arrows indicate the surrounding bone marrow. Scale bars in (ii): 100 µm.

Histological examination demonstrated direct bone-to-implant contact in both the uncoated and MC-coated groups, with no fibrous encapsulation (Fig. 6B and C). Furthermore, normal bone marrow structures were observed surrounding the implants in both groups, indicating bone healing and osseointegration.^51^ No infiltration of polymorphonuclear leukocytes (PMNs), macrophages, or lymphocytes was observed in both groups, suggesting the absence of inflammatory responses after 12 weeks of implantation.^52^ In addition, no evidence of foreign body reaction, tissue necrosis, or other adverse tissue changes was observed around the implants. These findings indicate biocompatibility with the host tissues and support the formation of a stable bone-implant interface. The absence of persistent inflammatory or foreign body responses further suggests osseointegration and maintenance of tissue homeostasis around both the uncoated and MC-coated implants. Previous studies have demonstrated that antimicrobial implant coatings can provide antibacterial functionality without compromising osseointegration, with coated implants exhibiting bone formation and bone-implant integration comparable to uncoated controls.^53^ Collectively, these results indicate that incorporation of MC within the PLA-NS coating did not compromise the biological processes required for successful osseointegration. These results indicate that the PLA-NS coating enabled local MC delivery while maintaining biological compatibility with the surrounding tissues. Future studies should evaluate the efficacy of the MC-coated dental implants in *in vivo* peri-implantitis models to determine whether sustained local MC delivery can effectively prevent peri-implant infection, inflammation, and associated bone loss under clinically relevant conditions.

## 4 Conclusion

In this work, we developed MC-coated dental implants using a PLA-NS coating system capable of continuous local antibiotic delivery. The coated implants released MC for at least 12 weeks, maintaining concentrations above the MIC for *P. gingivalis* and exhibiting antibacterial activity for up to 94 days. In addition, the MC-coated implants demonstrated good hemocompatibility and cytocompatibility *in vitro*. *In vivo* evaluations further confirmed their biocompatibility, with no evidence of irritation, sensitization, inflammatory responses, or adverse tissue reactions. Histological evaluation following 12 weeks of implantation further confirmed direct bone-to-implant contact, normal bone healing, and the absence of foreign body reactions, indicating that the coating did not adversely affect osseointegration. Collectively, these findings demonstrate that the PLA-NS coating enabled continuous local MC delivery while maintaining the biological performance required for dental implant applications. This approach opens opportunities for the development of drug-eluting dental implants and may facilitate the localized delivery of antimicrobial, anti-inflammatory, or regenerative agents to improve peri-implant tissue health and implant longevity.

## Author contributions

Napossorn Patiyananuwat: writing – review & editing, methodology, investigation, formal analysis. Chalaisorn Thanapongpibul: writing – original draft, formal analysis, visualization. Panarin Chinavinijkul: investigation. Bongkot Phumkrachai: investigation. Chinnadit Ngamwongronnachai: investigation. Salunya Tancharoen: conceptualization, supervision. Norased Nasongkla: writing – review & editing, conceptualization, supervision.

## Conflicts of interest

The authors declare that they have no known competing financial interests or personal relationships that could have appeared to influence the work reported in this paper.

## Data Availability

All data supporting the findings of this study are available within the article..
